# A peculiar low-luminosity short gamma-ray burst from a double neutron star merger progenitor

**DOI:** 10.1038/s41467-018-02847-3

**Published:** 2018-01-31

**Authors:** B.-B. Zhang, B. Zhang, H. Sun, W.-H. Lei, H. Gao, Y. Li, L. Shao, Y. Zhao, Y.-D. Hu, H.-J. Lü, X.-F. Wu, X.-L. Fan, G. Wang, A. J. Castro-Tirado, S. Zhang, B.-Y. Yu, Y.-Y. Cao, E.-W. Liang

**Affiliations:** 1 School of Astronomy and Space Science, Nanjing University, 210093 Nanjing, China; 2 Instituto de Astrofísica de Andalucía (IAA-CSIC), P.O. Box 03004, E-18080 Granada, Spain; 3 Key Laboratory of Modern Astronomy and Astrophysics, Nanjing University, Ministry of Education, Nanjing, 210093 China; 4 Department of Physics and Astronomy, University of Nevada, Las Vegas, NV 89154 USA; 5 Department of Astronomy, School of Physics, Peking University, 100871 Beijing, China; 6 Kavli Institute for Astronomy and Astrophysics, Peking University, 100871 Beijing, China; 7 National Astronomical Observatories, Chinese Academy of Sciences, A20 Datun Road, 100012 Beijing, China; 8 School of Physics, Huazhong University of Science and Technology, 430074 Wuhan, China; 9 Department of Astronomy, Beijing Normal University, 100875 Beijing, China; 10 Department of Space Sciences and Astronomy, Hebei Normal University, 050024 Shijiazhuang, China; 11 Purple Mountain Observatory, Chinese Academy of Sciences, 210008 Nanjing, China; 12 Department of Astronomy, University of Florida, 211 Bryant Space Science Center, Gainesville, FL 32611 USA; 13 Facultad de Ciencias, Campus Fuentenueva s/n, Universidad de Granada, E-18071 Granada, Spain; 14 Guangxi Key Laboratory for Relativistic Astrophysics, Department of Physics, Guangxi University, 530004 Nanning, China; 15 School of Astronomy and Space Science, , University of Science and Technology of China, 230026 Hefei, China; 16 School of Physics and Electronics Information, Hubei University of Education, 430205 Wuhan, China; 17 Gran Sasso Science Institute (INFN), Via Francesco Crispi 7, I-67100 LAquila, Italy; 18 INFN - Sezione di Pisa Edificio C, Largo Bruno Pontecorvo, 3, 56127 Pisa, Italy; 19 Departamento de Ingeniería de Sistemas y Automática, Escuela de Ingenierías, Universidad de Málaga, C. Dr. Ortiz Ramos sn, 29071 Málaga, Spain

## Abstract

Double neutron star (DNS) merger events are promising candidates of short gamma-ray burst (sGRB) progenitors as well as high-frequency gravitational wave (GW) emitters. On August 17, 2017, such a coinciding event was detected by both the LIGO-Virgo gravitational wave detector network as GW170817 and Gamma-Ray Monitor on board NASA’s *Fermi* Space Telescope as GRB 170817A. Here, we show that the fluence and spectral peak energy of this sGRB fall into the lower portion of the distributions of known sGRBs. Its peak isotropic luminosity is abnormally low. The estimated event rate density above this luminosity is at least $190_{ - 160}^{ + 440}$ Gpc^−3^ yr^−1^, which is close to but still below the DNS merger event rate density. This event likely originates from a structured jet viewed from a large viewing angle. There are similar faint soft GRBs in the *Fermi* archival data, a small fraction of which might belong to this new population of nearby, low-luminosity sGRBs.

## Abstract (web summary)

A short-duration gamma-ray burst was detected along with a double neutron start merger gravitational wave by LIGO-Virgo on August 17th 2017. Here, the authors show that the fluence and spectral peak energy of this event fall into the lower portion of the distribution of known short-duration gamma-ray bursts.

## Introduction

Short-duration gamma-ray bursts (sGRBs) have long been proposed to be produced in systems involving the coalescence of double neutron stars (DNS)^1^, and the observations of sGRB afterglows and host galaxies are consistent with such a conjecture^2–4^. Based on the estimated event rate density derived from previously observed sGRBs at cosmological distances^5, 6^, the chance of detecting an sGRB within a small volume for detectable DNS mergers by advanced LIGO is very low^7^. Thus, GRB 170817A^8^/GW 170817^9^, as the first event in history showing a GRB associated with a gravitation wave signal from a compact binary merger, provides a unique opportunity to study its event rate, merger product, and implications of the GRB physics.

In this work, we performed a comprehensive analysis on GRB 170817A, mainly focusing on its prompt emission data in *γ*-ray energy band. Taking NGC 4993 as its host galaxy^10^, we find that its luminosity is abnormally low. We calculate the event rate of such sGRB event rate density and performed a comparison between such a rate density and the NS–NS merger event rate density. We also discussed the possible jet geometries, the physical implication of the time delay between GW signal and GRB signal, and the possible merger products of the event.

## Results

### Light curve structure

**Fig. 1:**
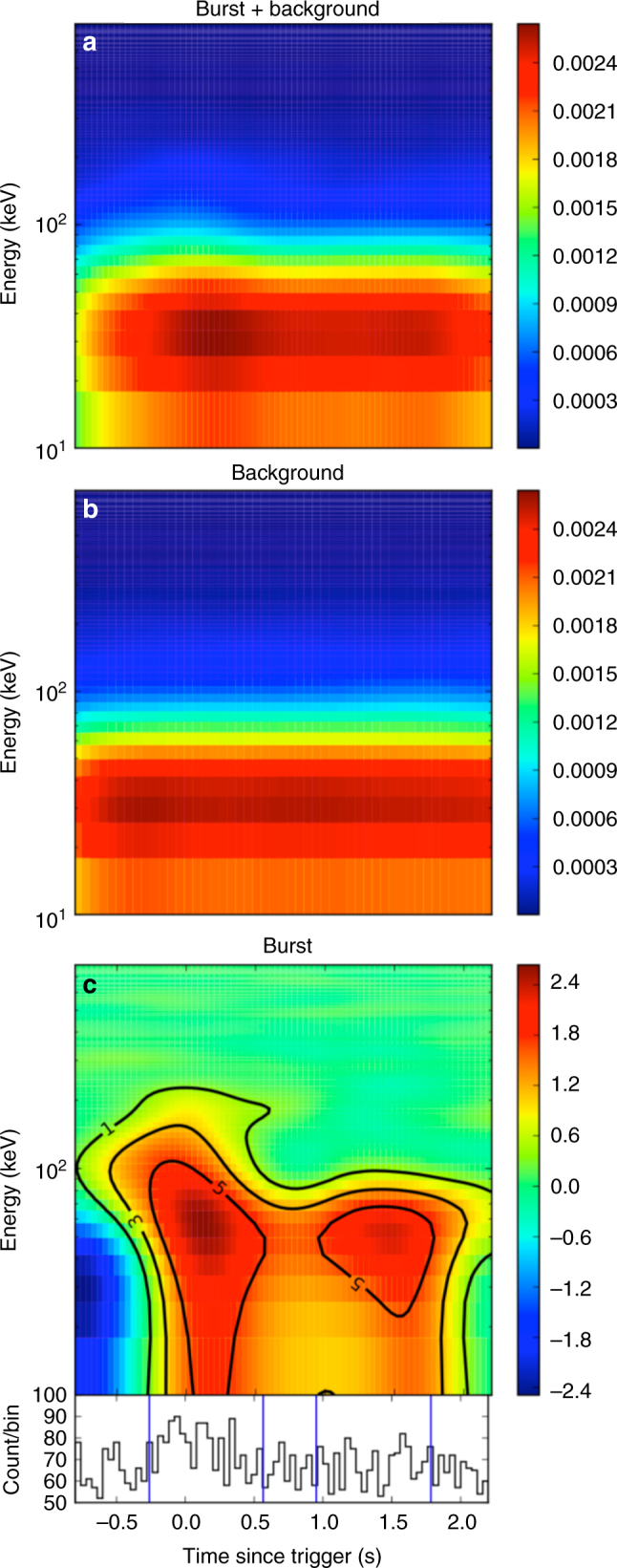
Signal detection from the *Fermi* GBM Time-Tagged Event (TTE) data of GRB 170817A. **a** The observed count map. **b** The count map in a background region. **c** The background-subtracted count map along with the 15–350 keV light curve. The contour lines represent the levels of signal-to-noise ratio

GRB 170817A (*Fermi* Trigger number 170817529) triggered Fermi GBM (8 keV–40 MeV)^11^ at *T*_0_ = 12:41:06.474598 UT on 17 August 2017^8^. We processed the public Fermi/GBM data using the procedure as described in ref. ^12^. We selected two GBM/NaI detectors, n1 and n2, on board *Fermi* that are in good geometric configurations (e.g., angle <60°) with respect to the source position. By extracting the photon events from the Time-Tagged Event (TTE) data detected by these two detectors, we noticed that a sharp peak is present in the light curve between *T*_0_ − 0.26 s and *T*_0_ + 0.57 s with a signal-to-noise ratio (S/N) >5 (Methods). Such a signal is clearly identified in the two-dimensional (2-D) count map presented in Fig. 1. A weaker tail, which is also significant above the background with S/N >5, appears between *T*_0_ + 0.95 s and *T*_0_ + 1.79 s. The total span of GRB 170817A is about 2.05 s with a 0.38-s gap consistent with the background. The burst was also detected in the data of the SPI Anti-Coincidence System (ACS) on-board International Gamma-Ray Astrophysics Laboratory (INTEGRAL)^13^. We downloaded the pre-binned (50-ms bin) SPI-ACS light curve from http://isdc.unige.ch/Soft/ibas/ibas_acs_web.cgi, which is derived from 91 independent detectors with different lower energy thresholds (from 60 to 120 keV) and an upper threshold of ~10 MeV. The multi-channel GBM light curves and the SPI-ACS light curves are presented in Supplementary Fig. 1. We performed an extended search for signals before and after the burst using GBM data and no significant emission was found (Supplementary Note 5; Supplementary Figs. 9 and 10).

### Spectral analysis

We first extract the time integrated spectrum in the first peak region (i.e., from *T*_0_ − 0.26 to *T*_0_ + 0.57 s). We select the NaI detectors n1 & n2 and BGO detector b0. The total number of photon counts is significantly above the background counts in NaI detectors (Supplementary Fig. 11). We used a software package developed by the first author, McSpecFit^14^, to perform spectral fitting. The energy channels at and around the iodine K-edge at 33.17 keV^15^ were excluded to better assess the quality of the fitting of spectral models. We find that the net spectrum can be successfully fitted by a power law function with an exponential high-energy cutoff (hereafter, cutoff power law or CPL model) with the goodness of statistics PGSTAT = 252.7 and degree of freedom DOF = 351 (Supplementary Note 1). The power law index is $- {\mathrm{0}}{\mathrm{.61}}_{ - 0.60}^{ + 0.34}$ and the cutoff energy, parameterized as *E*_p_, is ${\mathrm{149}}{\mathrm{.1}}_{ - 24.2}^{ + 229.4}$ keV. The corresponding average flux in this time interval is $2.19_{ - 0.62}^{ + 3.76} \times 10^{ - 7}$ erg cm^−2^ s^−1^ between 10 keV and 10 MeV. The fluence is $1.81_{ - 0.51}^{ + 3.11} \times 10^{ - 7}$ erg cm^−2^. For the second peak between *T*_0_ + 0.95 s and *T*_0_ + 1.79 s, we find that the net spectrum can be preferably fitted by a blackbody model with $kT = 11.3_{ - 2.4}^{ + 3.8}$ keV (PGSTAT/DOF = 236.4/352) although we cannot rule out its non-thermal origin due to the large uncertainty of the lower spectral index when fitted by a CPL model (Supplementary Table 1). The corresponding average flux in this time interval is $5.2_{ - 2.4}^{ + 4.7} \times 10^{ - 8}$ erg cm^−2^ s^−1^ between 10 keV and 10 MeV. The fluence in the same energy range is $4.33_{ - 1.99}^{ + 3.95} \times 10^{ - 8}$ erg cm^−2^. Including both peaks, the total fluence is $2.24_{ - 0.53}^{ + 3.51} \times 10^{ - 7}$ erg cm^−2^, corresponding to an isotropic energy of $4.17_{ - 0.99}^{ + 6.54} \times 10^{46}$ erg. Using a 50-ms time resolution light curve, the peak luminosity at *T*_0_ ≃ −0.07 s is derived as $1.6_{ - 0.4}^{ + 2.5} \times 10^{47}$ erg s^−1^. The best-fit parameters are presented in Supplementary Table 1. The spectral fitting plots as well as the parameter constraints are presented in Supplementary Figs. 2–4. No significant spectral evolution is observed (Supplementary Fig. 5).

### Spectral lag analysis

Using the cross correlation function (CCF) method, we also calculate the spectral lag of the GRB between (25–50) keV and (50–100) keV, which is 0.03 ± 0.05 s, consistent with zero. This is consistent with the spectral lag distribution of sGRBs^16^.

### Comparison with other GRBs

With the observed and derivative properties summarized in Table 1, one can compare GRB 170817A with other sGRBs. The following samples extracted from the Fermi/GBM catalog^17^ are considered for comparison: (a) the long GRB sample with *E*_p_ measured (1679 GRBs); (b) the short GRB sample (*T*_90_ < 2 s) with *E*_p_ measured (317 GRBs); and (c) the short GRB sample with S/N < 6 and *E*_p_ measured (66 “faint & short” GRBs). The latter is the faint sGRB sample to which GRB 170817A belongs (Supplementary Note 2 and Supplementary Fig. 6).

**Table 1:** Properties of GRB 170817A

Total spanning duration (s)	~2.05
Spectral peak energy (first peak) *E*_p_ (keV)	${\mathrm{149}}{\mathrm{.1}}_{ - 24.2}^{ + 229.4}$
Total fluence (erg cm^−2^)	$2.24_{ - 0.53}^{ + 3.51} \times 10^{ - 7}$
Spectral lag (25–50 keV vs. 50–100 keV)	0.03 ± 0.05 s
Redshift *z*	~0.009
Luminosity distance *D*_L_ (Mpc)	39.472
Total isotropic energy *E*_iso_ (erg)	$4.17_{ - 0.99}^{ + 6.54} \times 10^{46}$
Peak luminosity *L*_iso_ (erg s^−1^)	$1.6_{ - 0.4}^{ + 2.5} \times 10^{47}$

**Fig. 2:**
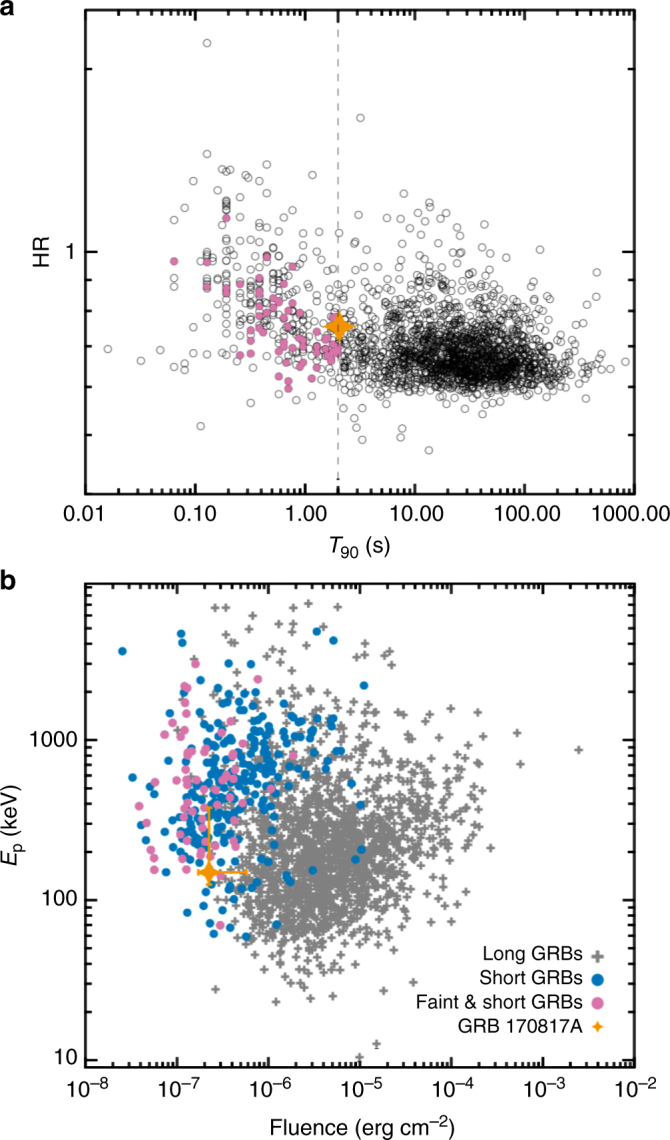
Comparisons between GRB 170817A and other GRBs. **a** A comparison between GRB 170817A and other Fermi long and short GRBs in the *T*_90_ − HR diagram. The hardness ratio (HR) is defined as ratio of the observed counts in the 50–100 keV band compared to the counts in the 25–50 keV band within the *T*_90_ region. **b** GRB 170817A in the fluence vs. *E*_p_ diagram against other sGRBs

We first compare the observed properties of GRB 170817A with other GRBs. Figure 2 upper panel is the standard *T*_90_ − HR (hardness ratio) plot for GRBs. One can see that GRB 170817A falls in the boundary between short and long GRB populations. Since evidence has suggested that majority of sGRBs are consistent with the compact star merger origin, GRB 170817A, being associated with GW170817, belongs to the long and soft regime of this population. Figure 2 lower panel compares GRB 170817A and other GRBs in the fluence vs. *E*_p_ diagram. GRB 170817A seems to lie far away from the majority of the long GRBs. Based on *γ*-ray information only, this burst would be more likely regarded as one of those normal (but faint and soft) short GRBs if there was no gravitational wave trigger. Comparing the host galaxy NGC 4993 of GRB 170817A with the host galaxies of other sGRBs^3, 18^, we find that NGC 4993 falls into the distribution of sGRB hosts in terms of half-light radius, stellar mass, and afterglow offset from the host galaxy (Supplementary Note 3; Supplementary Fig. 7).

**Fig. 3:**
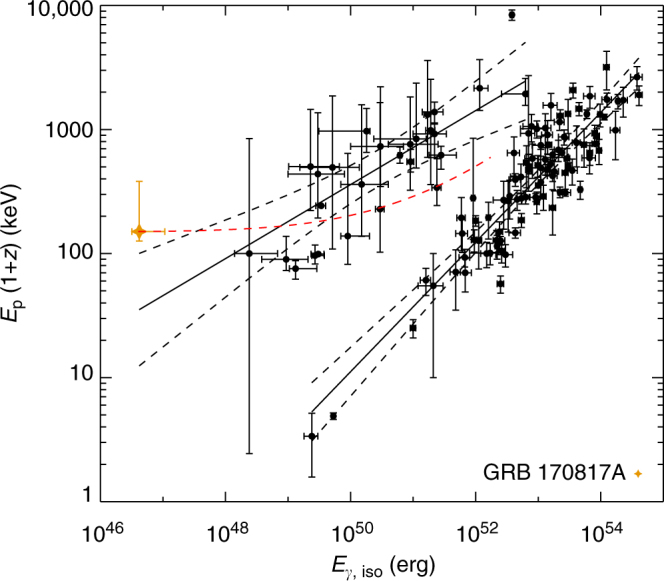
GRB 170817A in the *E*_p_ − *E*_iso_ correlation diagram. The solid lines are the best-fit correlations: log*E*_p_/(1 + *z*) = (3.24 ± 0.07) + (0.54 ± 0.04)log(*E*_iso_/10^52^) for short GRBs, log*E*_p_/(1 + *z*) = (2.22 ± 0.03) + (0.47 ± 0.03)log(*E*_iso_/10^52^) for long GRBs. Red dashed line represents GRB 170817A position if it were in different redshifts ranging from 0.009 to 3. All error bars represent 1−*σ* uncertainties

We next investigate the intrinsic property of the burst. Taking into consideration the very small distance *D*_L_ ~ 40 Mpc of the host galaxy NGC 4993^19^, this burst is abnormally low in terms of luminosity and energy (throughout the paper, luminosity and energy are the isotropic-equivalent ones). The peak isotropic luminosity with 50 ms bin size is $1.6_{ - 0.4}^{ + 2.5} \times 10^{47}$ erg s^−1^, and the isotropic energy is $E_{{\mathrm{iso}}} = 4.17_{ - 0.99}^{ + 6.54} \times 10^{46}$ erg. Such low-luminosity sGRBs have never been observed before. Plotting it onto the intrinsic peak energy *E*_p,*z*_ = *E*_p_(1 + *z*) vs. isotropic energy *E*_iso_ plane^20, 21^, we find that it is within the 2*σ* of the track of the sGRB population, but slightly deviates from the 1*σ* region of the track into the hard regime even if its *E*_p_ error is included (Fig. 3). The burst would be more normal if its isotropic luminosity was somewhat higher.

### Event rate density of the 170817A-like GRBs

Based on previously known sGRBs, the event rate density (also called volumetric event rate) of sGRBs above then-minimum luminosity (~10^50^ erg s^−1^) is a few Gpc^−3^ yr^−1 ^^5, 6^. For example, for a Gaussian distribution of the merger delay time^22^, the event rate density of sGRBs is $4.2_{ - 1.0}^{ + 1.3}$ Gpc^−3^ yr^−1^ above 7 × 10^49^ erg s^−1 ^^6^. This was significantly lower than the estimated DNS merger event rate density (ref. ^9^, see below). The discrepancy may be removed if one considers the beaming correction of sGRBs within the top-hat uniform jet model. Using the beaming factor *f*_b_ ~ 0.04 inferred from the sparse sGRB jet break data collected in the past^23^, the beaming-corrected event rate density (counting for sGRBs not beaming toward us) was ~100 Gpc^−3^ yr^−1^. With the detection of GRB 170817A, the distribution of the sGRB isotropic peak luminosity extended down by ~three orders of magnitude. The revised event rate density of sGRB above 1.6 × 10^47^ erg s^−1^ becomes (Methods)

1 $$\rho _{{\mathrm{0,sGRB}}}\left( {L_{{\mathrm{iso}}} > 1.6 \times 10^{47}\,{\mathrm{erg}}\,{\mathrm{s}}^{ - 1}} \right) = 190_{ - 160}^{ + 440}\,{\rm Gpc}^{ - 3}\,{\rm yr}^{ - 1}$$

if one assumes only one such event within the GBM archives. This is comparable to (or somewhat higher than) the previously derived beaming-corrected sGRB event rate density, but could be still up to a factor of a few smaller than the DNS merger event rate density derived based on the detection of GW 170817A^9^, which is (Methods)

2 $$\rho _{{\mathrm{0,DNS}}} = 1100_{ - 910}^{ + 2500}\,{\rm Gpc}^{ - 3}\,{\rm yr}^{ - 1}.$$

**Fig. 4:**
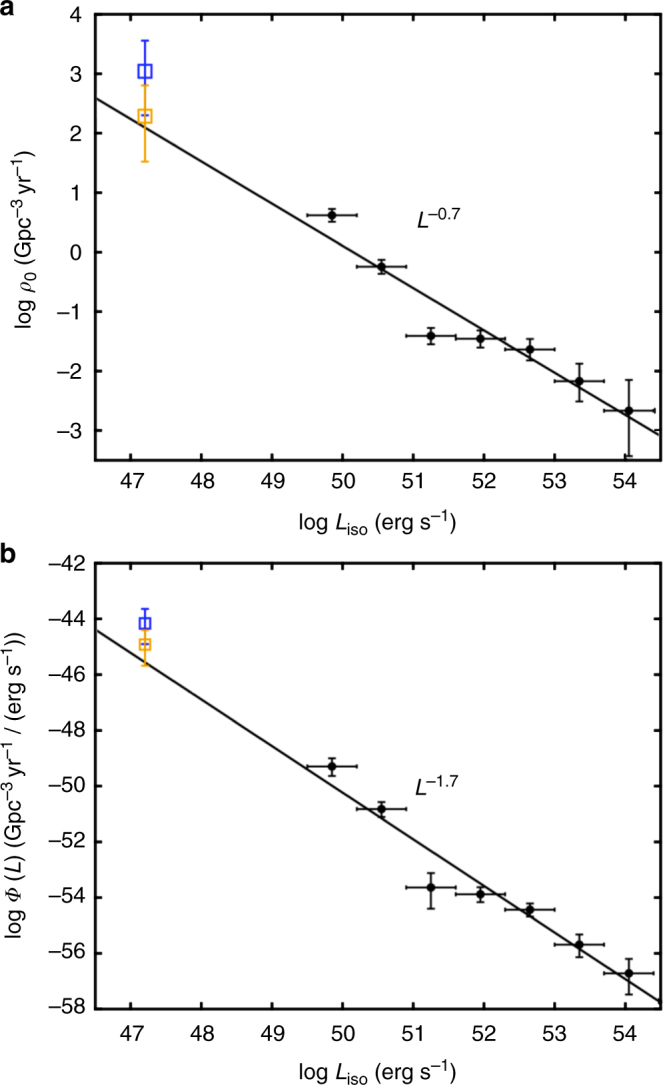
Distributions of local event rate density and luminosity function. **a** The local event rate density distribution of sGRBs including GRB 170817A. The orange symbol with error denotes the event rate density derived from GRB 170817A and the blue symbol with error denotes the DNS merger event rate density derived from GW170817. The black power-law line and other data points were derived from the *Swift* sGRB sample^6^. The vertical error bar represents the 1*σ* Gaussian errors derived from ref. ^35^. **b** Luminosity function distribution of sGRBs including GRB 170817A, with labels same as the upper panel. All error bars represent 1−*σ* uncertainties

Figure 4 upper panel shows the sGRB event rate density as a function of luminosity threshold. The black power-law (PL) line with an index −0.7 was derived from the *Swift* sGRBs (black crosses with error included, peak luminosity derived with 64 ms time bin) with redshift measurements^6^. GRB 170817A (orange) extends the sGRB luminosity by three orders of magnitude in the low-*L*_iso_ regime. Interestingly, the revised event rate density above 1.6 × 10^47^ erg s^−1^ follows the extension of the PL distribution derived by ref. ^6^. If one considers that there might be some sGRBs similar to GRB 170817A hidden in the GBM archives, the true event rate density could be higher, but has to be limited by the DNS merger event rate density (blue symbol). In Fig. 4 lower panel, we derive a new sGRB luminosity function across a wide range of luminosity, which is consistent with the extrapolation of the previous results that show a power law with $L_{{\mathrm{iso}}}^{ - 1.7}$^6^.

## Discussion

There are in principle two possibilities to produce a low-luminosity sGRB from a DNS merger. The first possibility is a bright sGRB jet viewed off-axis. Within this picture, the main jet (similar to the one observed from a more distant sGRB) beams toward a different direction. However, within such a scenario, one cannot have a sharp-edge conical jet viewed outside the jet cone. This is because the observed duration would be longer than the central engine activity time scale, inconsistent with its typical sGRB duration (Supplementary Note 6). Rather, one requires a structured jet viewed from a large wing^24, 25^ with emission powered by the low-luminosity wind along the line of sight. Within the sGRB context, such a jet configuration has been discussed in terms of a jet-cocoon geometry^26, 27^. A viewing angle *θ*_v_ ≤ 28° (or ≤36° depending on the assumed value of the Hubble constant^28^) has been inferred from the gravitational wave data. This is consistent with such a scenario. The second possibility is that the outflow of GRB 170817A may have an intrinsically low luminosity. However, the late rise of X-ray and radio flux^29, 30^ from the source suggests that the total energy budget of the source is higher. It disfavors this second possibility but favors the off-axis structured jet scenario (Supplementary Note 9).

The short duration of the burst is consistent with a prompt black hole or a hyper-massive neutron star that survived for a short (e.g., ~100 ms) period of time before collapsing to a black hole. We conduct a search of precursor or extended emission before and after the GRB trigger time and give a negative result (Supplementary Note 5). Even though the possibility of a long-live post-merger neutron star product cannot be ruled out from the GW^31^ and EM data, our non-detection of extended *γ*-ray emission is consistent with a BH post-merger product (Supplementary Note 7).

The merger time of the gravitational wave signal is at *T*_GW_ = 12:41:04.430 ± 0.002 UTC on 17 August 2017 (GPS time *T*_GW_ = 1187008882.430 ± 0.002 s)^28^. The beginning time of GRB 170817A (~ −0.3 s with respect to the *Fermi*/GBM trigger time *T*_0_ = 12:41:06.474598 UT on 17 August 2017^8^) has an ~1.7-s delay with respect to the merger time. It is intriguing that this delay time scale has the same order as the burst duration itself. Such a delay offers a diagnostic of the emission site and energy dissipation process of GRBs. In particular, a scenario that invokes a magnetized jet dissipating in an optically thin region can interpret both time scales simultaneously without introducing an ad hoc jet-launching delay time as most other models do (Supplementary Note 8).

Assuming a standard radiative efficiency and standard shock microphysics parameters, the low isotropic energy of GRB 170817A suggests that the multi-wavelength afterglows of the burst should be very faint (Supplementary Note 6). We used the Javier Gorosabel 0.6 m robotic telescope at the BOOTES-5 station at Observatorio Nacional de San Pedro Martir (Mexico) to image the 15 galaxies in the GLADE Catalogue starting on Aug 18.21 UT. The optical counterpart (SSS17a) of GW 170817 was detected in the outskirts of the NGC 4993 galaxy, with a magnitude *R* = 18.20 ± 0.45, in agreement with other contemporaneous measurements. This is much brighter than the predicted flux of optical afterglow. As a result, this optical transient originates from a quasi-thermal kilonova^32, 33^, as suggested by independent modeling of many authors (e.g., ref. ^34^).

Within the Fermi GBM soft faint sGRB sample, there might be at most GRB 170817A-like events limited by the DNS merger rate. Some short, faint sGRB events are presented in Supplementary Fig. 6. However, identifying them turns out difficult without gravitational wave detections (Supplementary Note 10).

## Methods

### Determining GRB duration using 2-D energy vs. time count map

The GRB duration is usually defined by *T*_90_, which is the time span over which 5–95% of its total measured counts are measured^17^. The calculation of *T*_90_ is subject to the selection of energy band, the bin-size as well as the assumption of the model background (e.g., a 2nd or 3rd order polynomial function) of the GRB light curve. To minimize such an artificial effect for a faint GRB like GRB 170817A, we utilize the 2-D count map of the photon energy and photon arrival time directly from the *Fermi*/GBM TTE data and calculate the GRB duration. Our procedure is the following: (1) select the source region between the time interval [t1, t2] that includes the GRB signal. For GRB 170817A, [t1, t2] = [−1, 3]. (2) Select all the photon events between [t1, t2] in the *Fermi*/GBM TTE data. Note the selected data are a list of [time, energy] pairs. (3) Convert time vs. energy pairs to 2-D points in the time vs. energy plot, then use Kernel Density Estimation (KDE) to plot a 2-D source count map in the time vs. energy plot. This is the top panel in Fig. 1. (4) Select two background regions [t3, t4] and [t5, t6] that are before and after the burst region. For GRB 170817A, [t3, t4] = [−10, −2] and [t5, t6] = [5, 10]. Repeat steps (2) and (3) to get two 2-D count maps for the pre-burst and after-burst backgrounds. (5) Perform interpolation between those two background count maps to calculate the source-normalized background count map within [t1, t2] (middle panel of Fig. 2). Such a normalized and interpolated background within the source region can be used to calculate the standard derivation (STD) of the background. (6) Subtract the background count map from the source count map to get the net count map (bottom panel of Fig. 1). For each bin in the count map, define its signal-to-noise ratio as S/N = (net count)/STD. Overplotting the S/N = 1, 3, 5 in the net count map, we then define the burst duration region as where S/N ≥ 5.0 is satisfied.

### sGRB event rate density

The abnormally low luminosity and extremely small distance of GRB 170817A suggest that the actual event rate density of short GRBs is large. With one detection, one can estimate the local event rate density *ρ*_0,sGRB_ of short GRBs through

3 $$N_{{\mathrm{sGRB}}} = \frac{{{\mathrm{\Omega }}_{{\mathrm{GBM}}}T_{{\mathrm{GBM}}}}}{{4\pi }}\rho _{{\mathrm{0,sGRB}}}V_{{\mathrm{max}}} = 1.$$

The field of view of GBM is approximatively taken as full sky with ${\mathrm{\Omega }}_{{\mathrm{GBM}}} \simeq 4\pi$. The working time of GBM is taken since 2008 with a duty cycle of ~50%, so that $T_{{\mathrm{GBM}}} \simeq 4.5\,{\mathrm{yrs}}$. The maximum volume a telescope can detect for this low-luminosity event is $V_{{\mathrm{max}}} = 4\pi D_{{\mathrm{L,max}}}^3{\mathrm{/}}3$. We simulate a set of pseudo-GRBs by placing GRB 170817A to progressively larger distances, and find that the signal would not be detectable at 65 Mpc (Supplementary Note 4; Supplementary Fig. 8). Taking this distance as *D*_L,max_, we derive the event rate density of sGRBs^6^

4 $$\rho _{{\mathrm{0,sGRB}}}\left( {L_{{\mathrm{iso}}} > 1.6 \times 10^{47}\,{\mathrm{erg}}\,{\mathrm{s}}^{ - 1}} \right) = 190_{ - 160}^{ + 440}\,{\mathrm{Gpc}}^{ - 3}\,{\mathrm{yr}}^{ - 1},$$

assuming only one such sGRB exists in the GBM archives. This number may be regarded as a lower limit if in reality there are other hidden ones.

The event rate density of DNS mergers may be also estimated based on one detection by aLIGO during O1 and O2. Since only one DNS merger event was detected^9^, one may write

5 $$N_{{\mathrm{DNS}}} = \frac{{{\mathrm{\Omega }}_{{\mathrm{LVC}}}}}{{4\pi }}\rho _{{\mathrm{0,DNS}}}\left( {V_{{\mathrm{max,O1}}}T_{{\mathrm{O}}1} + V_{{\mathrm{max,O2}}}T_{{\mathrm{O}}2}} \right) = 1.$$

Noticing Ω = 4*π* for GW detectors, taking DNS merger horizon ~60 and ~80 Mpc for O1 and O2, respectively, and adopting a duty cycle of ~40% for both O1 and O2, we estimate

6 $$\rho _{{\mathrm{0,DNS}}} = 1100_{ - 910}^{ + 2500}\,{\mathrm{Gpc}}^{ - 3}\,{\mathrm{yr}}^{ - 1}.$$

This is consistent with the DNS merger event rate density derived by the LIGO-VIRGO team using more sophisticated simulations^9^.

The error bars in both Eqs. (4) and (6) show the 1*σ* Gaussian errors derived from ref. ^35^ by taking only one observational event into account. Comparing the two equations, one can see that even though the sGRB rate density may be consistent with the DNS merger rate density, it can be smaller than the latter by up to a factor of a few. This either suggests that there might be even less luminous sGRBs than GRB 170817A, or there might be GRB 170817A-like sGRBs hidden in the GBM archives. The number of these events is at most a few.

### Data availability

The data that support the plots within this paper and other findings of this study are available from the corresponding author upon reasonable request.

## Electronic supplementary material

Supplementary Information

Peer Review File
