# Supplementary material for: A peculiar low-luminosity short gamma-ray burst from a double neutron star merger progenitor
Source: Nat Commun. 2018 Jan 31;9:447. doi: 10.1038/s41467-018-02847-3 (PMC5792494; doi:10.1038/s41467-018-02847-3)
Supplement: Supplementary file 1 — Supplementary Information [file 41467_2018_2847_MOESM1_ESM.pdf]

Supplementary Table I: Spectral Fitting Results of GRB 170817A

Time	Cutoff Power-Law Fitting				Power-Law Fitting			Blackbody Fitting			Model Comparison	
t1 ∼ t2 (s)	α	E_p (keV)	$\frac{PGSTAT}{dof}$	BIC	α	$\frac{PGSTAT}{dof}$	BIC	kT (keV)	$\frac{PGSTAT}{dof}$	BIC	BIC_{pl} - BIC_{cpl}	BIC_{bb} - BIC_{cpl}
-0.26 ∼ 0.57	$-0.61^{+0.34}_{-0.60}$	$149.1^{+229.4}_{-24.2}$	252.7/351	270.29	$-1.61^{+0.09}_{-0.13}$	261.9/352	273.68	$29.0^{+10.5}_{-8.5}$	261.1/352	272.83	3.38	2.53
-0.3 ∼ 0.05	$0.07^{+0.72}_{-0.92}$	$147.9^{+160.7}_{-28.4}$	237.1/352	254.70	$-1.65^{+0.12}_{-0.44}$	248.0/352	259.71	$34.6^{+13.6}_{-7.3}$	238.2/352	249.89	5.01	-4.8
0.05 ∼ 0.4	$-0.78^{+1.01}_{-0.84}$	$62.4^{+77.5}_{-21.8}$	209.1/351	226.77	$-2.12^{+0.22}_{-0.52}$	211.9/352	223.66	$10.5^{+12.2}_{-2.1}$	211.2/352	222.97	-3.11	-3.79
0.95 ∼ 1.79	$2.65^{+0.23}_{-3.66}$	$42.5^{+28.2}_{-11.1}$	236.3/351	253.90	$-2.27^{+0.63}_{-2.86}$	241.4 /352	253.19	$11.3^{+3.85}_{-2.36}$	236.35/352	248.09	-0.72	-5.81

Supplementary Note 1. Detailed light curves and spectral fit to GRB 170817A

The multi-channel light curves observed by Fermi/GBM and INTEGRAL/SPI-ACS is presented in Supplementary Figure 1.

As discussed in the main text, a cutoff power law presents an adequate fit to the spectral data. The best fitting result for the time interval (T_0-0.26,T_0+0.57) is presented in Supplementary Figure 2. We noticed that a simple power law model can also fit the data with a power law index $-1.61^{+0.09}_{-0.13}$ and PGSTAT/dof 261.9/352. To check whether the cutoff power law fit is overfitting (since it has one extra parameter), we employ a Bayesian information criterion (BIC) [1] to check its statistical confidence. As shown in Supplementary Table I, the comparison of the two models leads to ΔBIC =3.38 (cutoff power law model has the lower BIC). As suggested by [1], such a ΔBIC value indicates positive evidence against the model with a higher BIC (Power Law model in this case). So we favorably choose the cutoff power law model throughout our analysis.

We also compare the fits between the blackbody (BB) model and the CPL model. For the time-integrated spectral fitting, according to BIC, the BB model is less preferred to fit the observed spectra. We also notice the best-fit low energy photon index -0.66 in the CPL model is too soft to match the blackbody value (+1).

According to BIC, the weaker emission between 0.95 s and 1.79 s is favorably fitted by the BB model with $kT = 11.3^{+3.85}_{-2.36}$ keV. The best fitting result of this time interval is presented in Supplementary Figure 3.

Due to the low number of photon counts and short duration, the finest bin size we are able to perform a time-resolved spectral analysis is around 0.2-0.3 s, below which the spectral parameters become unconstrained. We select the brightest region between $T_0 - 0.3$ s and $T_0 + 0.4$ s in the first peak, divide it into two equal slices, and perform the time-dependent spectral analysis on them. We present the spectral evolution properties in Supplementary Table I and Supplementary Figure 4. Our analysis suggests that there are indeed some spectral differences between the two slices.

Supplementary Note 2. Definition of The Faint Short GRB Sample

In Supplementary Figure 6, we plot all the Fermi GBM short GRBs in terms of their signal-to-noise ratio (S/N), fluence, and E_p. The bin where GRB 170817A is located in is marked as the red dashed vertical line. The sample to the left of the line in the S/N plot is defined as the faint sGRB sample. We plot some examples of the light curves of this sample in Supplementary Figure 6.

Supplementary Note 3. NGC 4993 as a sGRB host

The host galaxy NGC 4993 of GRB 170817A is an elliptical galaxy in the constellation Hydra. In Supplementary Figure 7 left panel we plot the half light radius R_{50} and stellar mass M_* of NGC 4933 against those of other short GRBs ([2] and references therein). It is found that NGC 4933 falls in the middle of the distributions and can be regarded as a typical short GRB host. The optical transient SSS17a has a projected distance of 10.6" from the center of NGC 4993. We plot the physical and normalized offset of this event and compare it with other sGRBs (Supplementary Figure 7 right panel). Again, it is consistent with other sGRBs.

Supplementary Note 4. The maximum detectable distance of GRB 170817A

In order to check at what distance GRB 170817A will become undetectable, we simulate several light curves in 15-350 keV by placing the burst at progressively larger distances from 45 to 80 Mpc. The background level is assumed to be unchanged. The source count rate is assumed to scale as $D_{\rm L}^{-2}$. A 1-σ Poisson noise was added in each simulation. Our simulation suggests that the burst would become hardly detected at $D_{\rm L} \simeq 65$ Mpc (Supplementary Figure 8). We therefore adopt this value as $D_{\rm L,max}$ to estimate the event rate density of GRB 170817A-like GRBs.

Supplementary Note 5. Amplitude parameter and possible underlying emission

The authors of Ref. [3] defined an amplitude parameter f, which is the ratio between the peak flux and average background flux of a burst. They found that the f parameter can be used to search for disguised short GRBs due to the "tip-of-iceberg" effect. Arbitrarily raising the background flux, one can always reduce the duration of a long GRB until its measured duration is shorter than 2 s. The amplitude parameter for such a pseudo GRB was defined as $f_{\rm eff}$ in [3]. Comparing the f values of short GRBs and $f_{\rm eff}$ values of long GRBs, [3] found that most short GRBs have an f value that is large enough (say, above 2) so that they are genuine. Performing the same analysis to GRB 170817A, we find that its amplitude parameter is relatively small, i.e. $f \sim 1.43$. As shown in Supplementary Figure 9, this value (red star) is smaller than most short GRBs, and may be confused as a disguised sGRB. The probability (p) for it to be a disguised sGRB is $p \sim 0.17$ according to the $p - f$ relation derived by [3]. The probability that this burst is an intrinsically short GRB is higher than being a disguised sGRB. Nonetheless, the probability that the intrinsic duration is long is not negligible. One cannot rule out the possibility that there is an underlying, weak, long-duration emission component below the background. In order to search for a possible signal before and after the burst, we perform a detailed spectral analysis in the following time intervals: -2.0 ∼ -1.4, -1.4 ∼ -0.8 , 4.0 ∼ 13.2 , 13.2 ∼ 22.4, 22.4 ∼ 31.6, 31.6 ∼ 40.8, and 40.8 ∼ 50.0 seconds. We did not find any significant emission above the background in any of these time intervals and the spectral fitting performed in these intervals simply give overfit/unconstrained parameters.

Supplementary Note 6. Off-axis model

For a uniform jet with a sharp edge viewed outside the jet cone, the observed duration is different from the observed duration of an on-beam observer, given the same central engine activity time scale. The ratio between the two times reads [4]

$$\frac{t(\rm off-beam)}{t(\rm on-beam)} = \frac{\mathcal{D}(\theta=0)}{\mathcal{D}(\theta=\theta_v-\theta_j)} = \frac{1-\beta\cos(\theta_v-\theta_j)}{1-\beta}, \tag{1}$$

where θ_j is the jet opening angle, θ_v is the viewing angle from the jet axis, $\mathcal{D} = 1/\Gamma(1-\beta\cos\theta)$ is the Doppler factor, β is the dimensionless velocity, Γ is the Lorentz factor, and θ is the angle from the line-of-sight. Since the on-beam Doppler factor can be much larger than the off-beam one, the off-beam duration can be much longer than the on-beam on. The observed ~ 2.05 s duration falls into the distribution

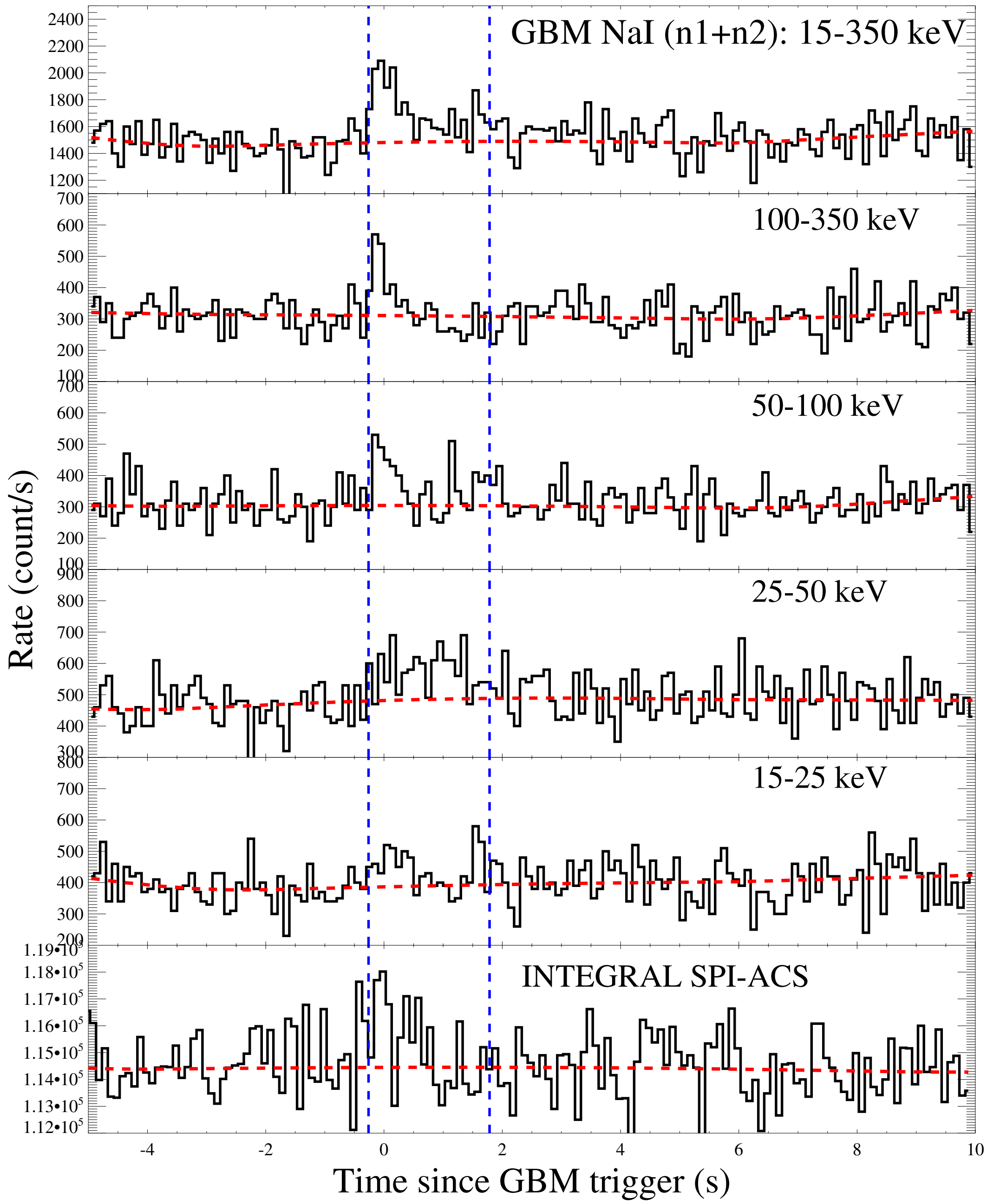

Supplementary Figure 1: Multi-Channel Light Curves observed by *Fermi*/GBM and *INTEGRAL*/SPI-ACS. The two vertical dashed lines indicate the S/N>5 region as shown in Figure 1.

of sGRBs. This limits the possible viewing angle of the top-hat model to be at most slightly outside the jet cone. This would suggest that the on-beam luminosity not much brighter than the observed luminosity. This is in conflict with the late time X-ray [5] and radio [6] data, which require significant energetics in the jet axis direction. We therefore conclude that the top-hat jet model viewed outside the jet cone is disfavored by the data.

A more reasonable model invokes a structured jet with angle-dependent isotropic luminosity, with the line of sight piecing through a low-luminosity wing of the jet along which there still exists a relativistic outflow towards the observer. The luminosity structure of the jet can be a simple function (e.g. power law or Gaussian) of the polar angle θ [7, 8] or a more complicated function (e.g. two or more components. Within the NS-NS merger scenario, a two-component scenario may be relevant, which invokes a narrow jet beam and a surrounding cocoon material formed to as the jet penetrates through the surrounding dynamical ejecta [9, 10]. In any case, the observed duration in a structured jet scenario is defined either by the central engine activity time scale, or the time scale during which the ejecta

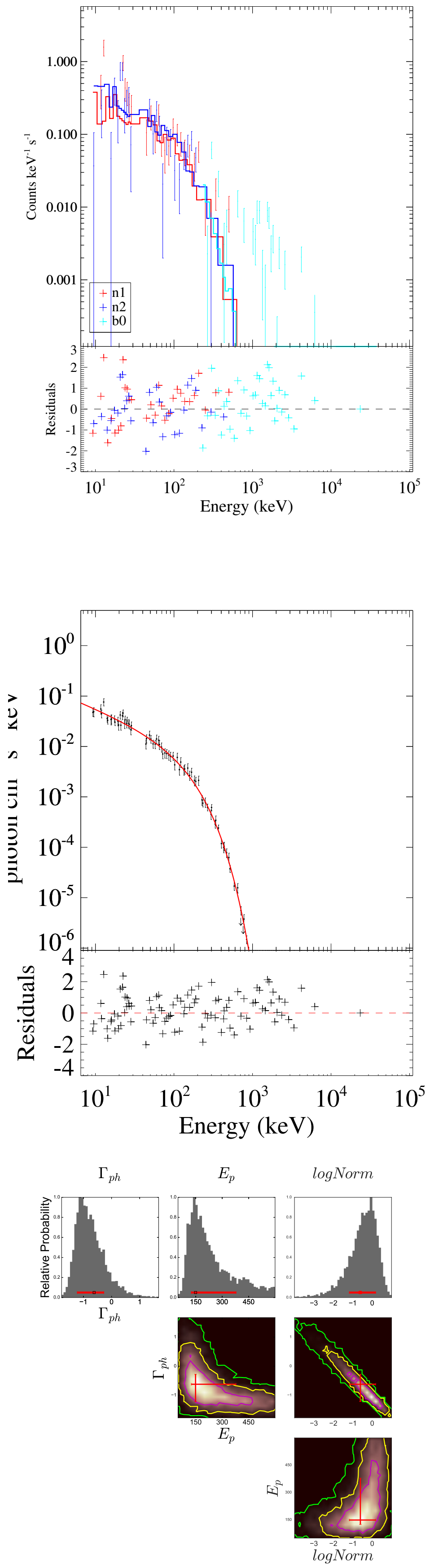

Supplementary Figure 2: Spectral fitting result for the interval ($T_0 - 0.26$, $T_0 + 0.57$). *a*: count spectrum. *b:* de-convolved photon spectrum. *c*: parameter likelihood map. All error bars represent 1-σ uncertainties.

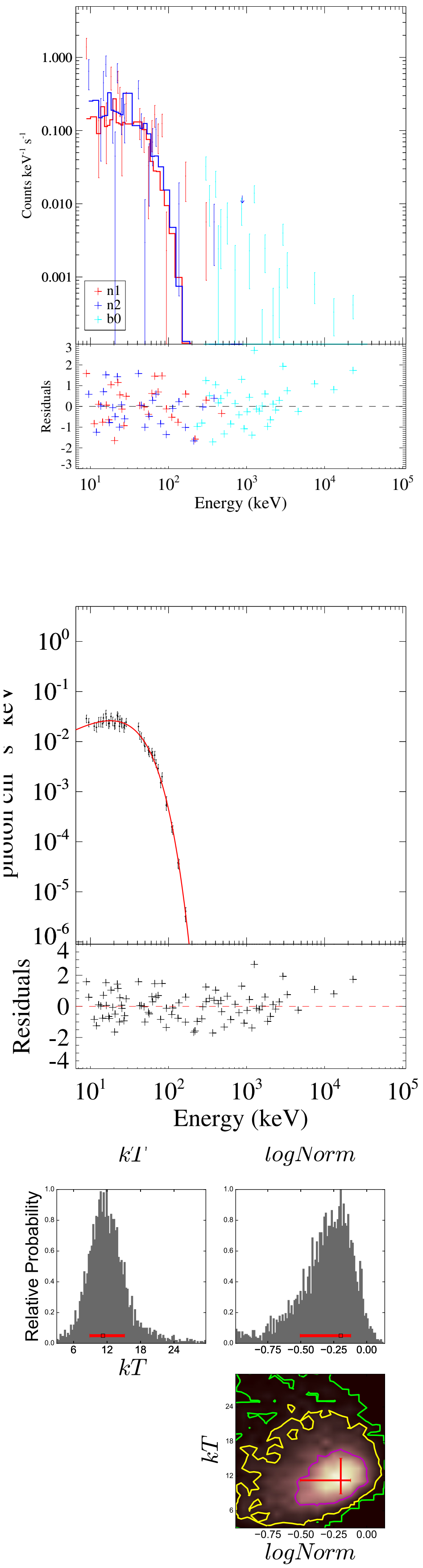

Supplementary Figure 3: Spectral fitting result for the interval $(T_0 + 0.95, T_0 + 1.79)$ s. *a*: count spectrum. *b*: de-convolved photon spectrum. *c*: parameter likelihood map. All error bars represent 1-σ uncertainties.

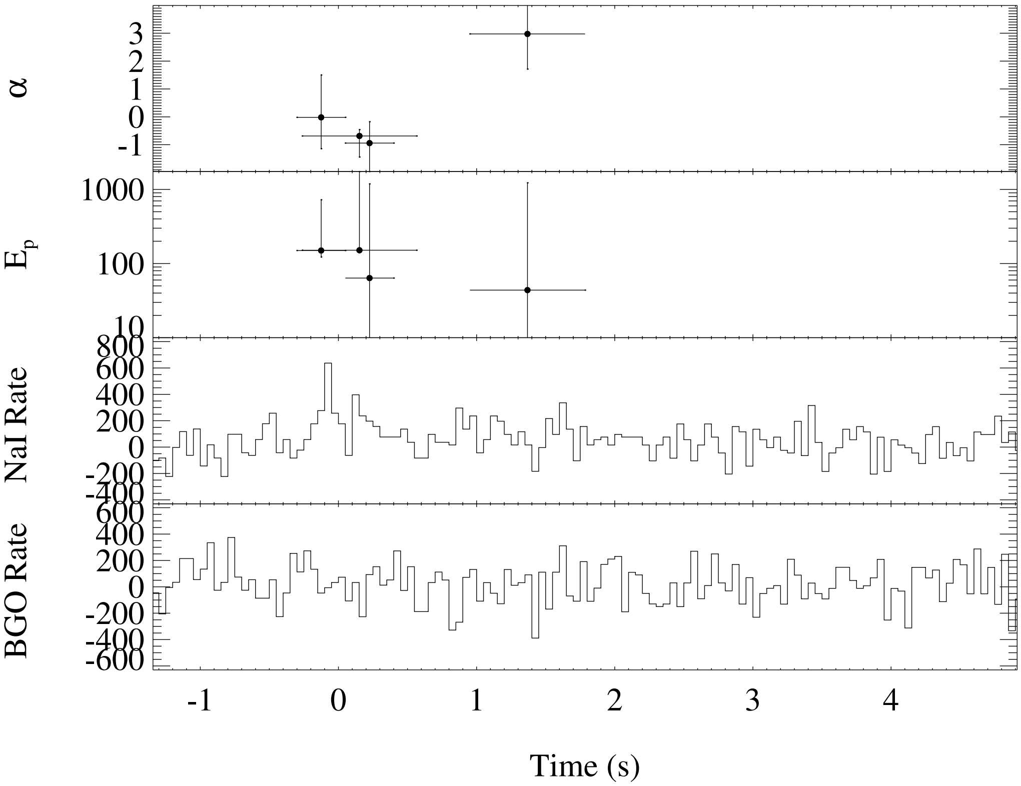

Supplementary Figure 4: Spectral evolution of the burst. Top two panels show evolution of the photon index (α) and peak energy (E_p). The time intervals are listed in Supplementary Table 1. Bottom two panels show the NaI (n1) light curve in 15-350 keV and BGO light curve in 250- 20000 keV. All error bars represent 1-σ uncertainties.

radiates, similar to the on-beam case. Invoking a structured jet viewed at a large angle from the jet axis (where the luminosity and bulk Lorentz factor may be both low), one can naturally interprets the duration and low-luminosity of the burst as well as the delayed onset of X-ray and radio emission from the source (e.g. [11]).

Supplementary Note 7. Merger product

The null result of our search for precursor and extended emission before and after the sGRB 170817A is consistent with a BH central engine. Since GW and EM data cannot rule out the possibility of a long-lived neutron star product (e.g. [12]), one may use the available data to constrain the parameter of the underlying NS. Neglecting gravitational wave spindown, the initial dipole spindown luminosity (e.g. [13]) $L_{\rm sd} = 10^{47}$ erg s$^{-1}(B_{p,14}^2 P_{0,-3}^{-4} R_6^6)$ can be constrained by the sGRB luminosity itself. Here B_p is the surface magnetic field at the polar region, P_0 is the initial rotation period, and R is the radius of the pulsar, and the convention $Q = 10^n Q_n$ has been adopted in cgs units. Writing $L_\gamma = \eta_\gamma L_{\rm sd}$ where η_γ is an efficiency parameter, in order to satisfy the luminosity constraint, the pulsar needs to have a magnetic field strength $B_p < 7.3 \times 10^{13}$G $P_{0,-3}^2 \eta_\gamma^{-1/2}$. On the other hand, the energy budget of the kilonova and afterglow places a significant limit on the total energy budget of the neutron star. The product has to be either rotating very slowly (which is inconsistent with the expectation of a merger), or has a very low magnetic field together with significant gravitational wave loss more than previously estimated [14, 15] (see a detailed analysis by [16]). We therefore suggest that a long-lived neutron star product is less preferred than a BH post-merger product.

Supplementary Note 8. Delay time between the γ-ray event and the gravitational wave event

The merger time derived from the GW signal is T_{GW}=12:41:04.4 UTC on 17 August 2017[35], which leads the GRB beginning time by $\Delta t \simeq 1.7$ s. This delay poses interesting constraints on the GRB emission models.

For a merger that produces a BH, a jet is likely launched promptly after the merger. This is certainly the case for a prompt BH formation, but would also likely be the case even if there is a short hypermassive neutron star (NS) phase. Even if the system would hold on launching the jet (say, by $\Delta t_{\rm jet}$), the time scale for a hypermassive NS is typically much shorter than 1.7 s (e.g. 100 ms).

The BH accretion time scale is short. The fall-back time reads

$$t_{\rm fb} \simeq 2 \left(\frac{R_{\rm out}^3}{GM_\bullet} \right)^{1/2}, \quad (2)$$

where $R_{\rm out}$ is the outer edge of disk. Usually, we assume the accretion begins at $R_{\rm out} \simeq 2R_{\rm T}$, where $R_{\rm T}$ is the tidal disruption radius

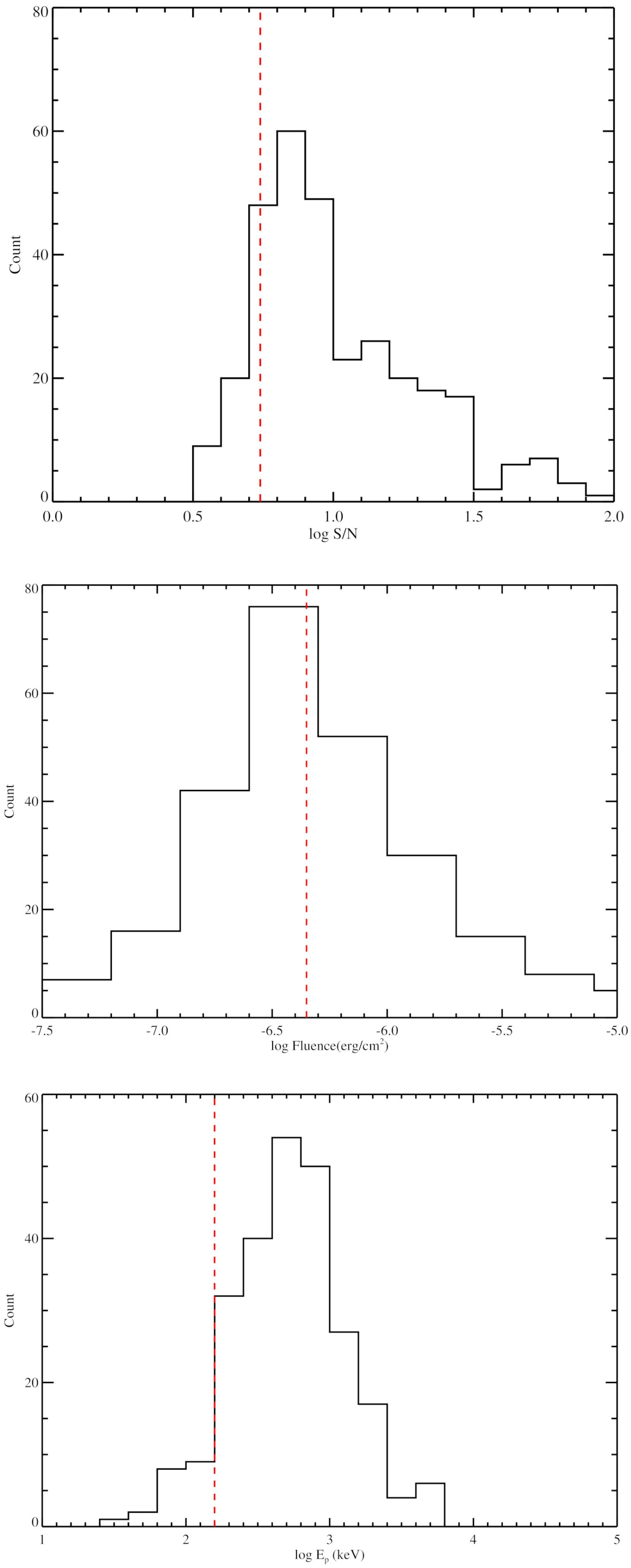

Supplementary Figure 5: The Fermi GBM short GRB histograms in terms of S/N ratio distribution, fluence distribution, and E_p distribution. The vertical red lines indicate the values for GRB 170817A.

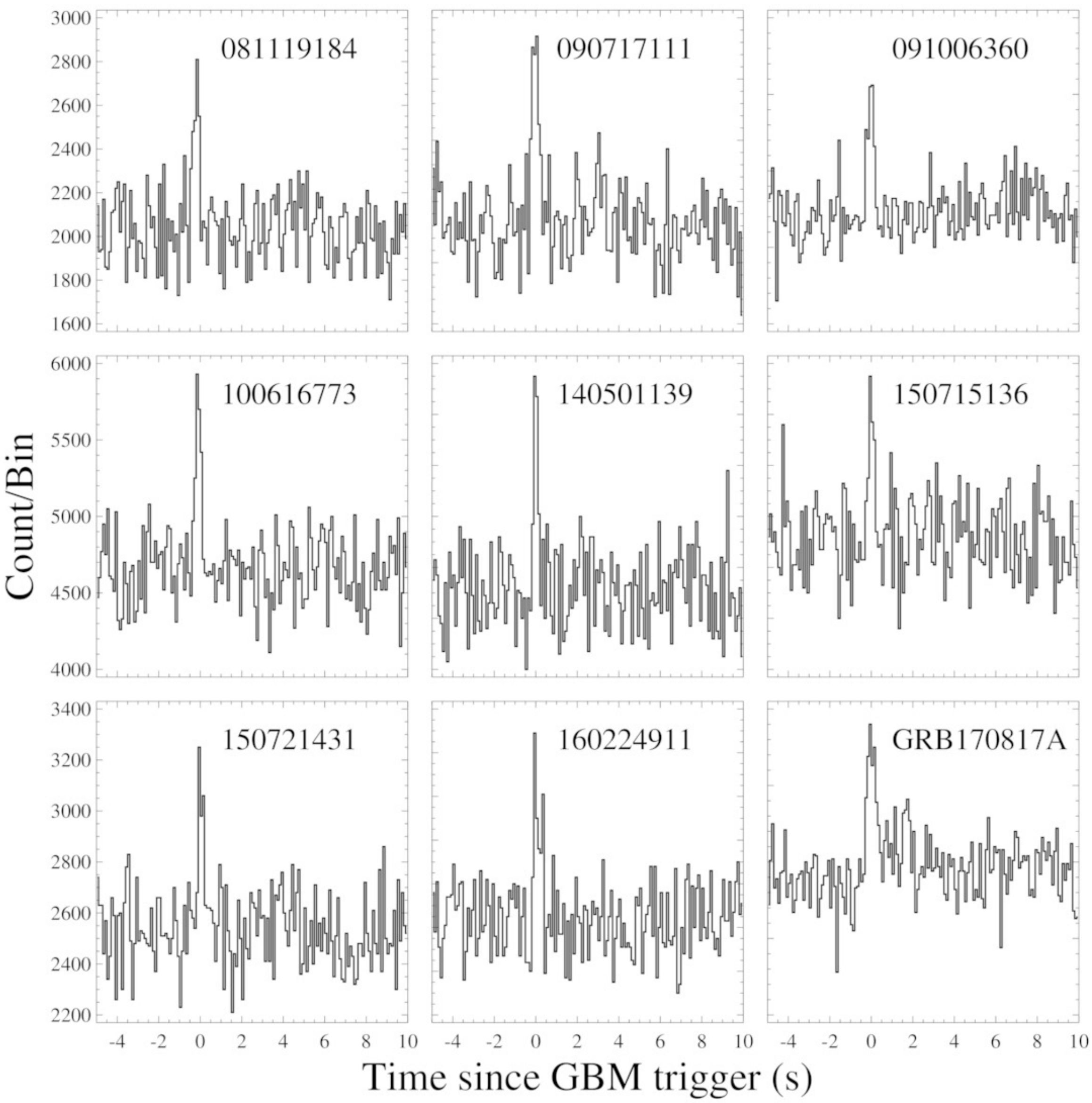

Supplementary Figure 6: Light curve examples for other GRB 170817A-like events.

with $M_\bullet/R_{\rm T}^3 \sim 4.2\rho_{\rm NS}$. One thus finds,

$$t_{\rm fb} \simeq 2\left(\frac{2}{G\rho_{\rm NS}}\right)^{1/2} \simeq 5\times10^{-4}s, \tag{3}$$

in which we adopt the typical density for neutron star (NS), i.e., $\rho_{NS} \sim 4\times10^{14}$g cm^{-3}.

A more relevant timescale is the accretion timescale, which may be estimated as

$$\tau_{\rm acc} \simeq \frac{t_{\rm fb}}{\alpha} \simeq 5\times10^{-3}\left(\frac{\alpha}{0.1}\right)\,{\rm s}, \tag{4}$$

where α is the viscosity parameter. One can see that this time scale is still much shorter than the observed delay.

A longer time scale comes from the propagation of the jet before releasing γ-rays. The delay time scale depends on the distance of the emission region from the central engine. In GRB models, both photosphere emission (which corresponds to a small radius $R_{ph} = L_w\sigma_{\rm T}/(8\pi\Gamma^3 m_p c^3) \simeq (5.9\times10^{10}$ cm) $L_{w,47}^{1/2}\Gamma_1^{-3}$, where $L_w = 10^{47}$ erg s$^{-1}L_{w,47}$ is the wind luminosity, and $\Gamma = 10\Gamma_1$ is the Lorentz factor of the flow) and synchrotron radiation from an optically thin region ($R_{\rm GRB} \gg R_{ph}$) have been invoked to interpret GRB emission.

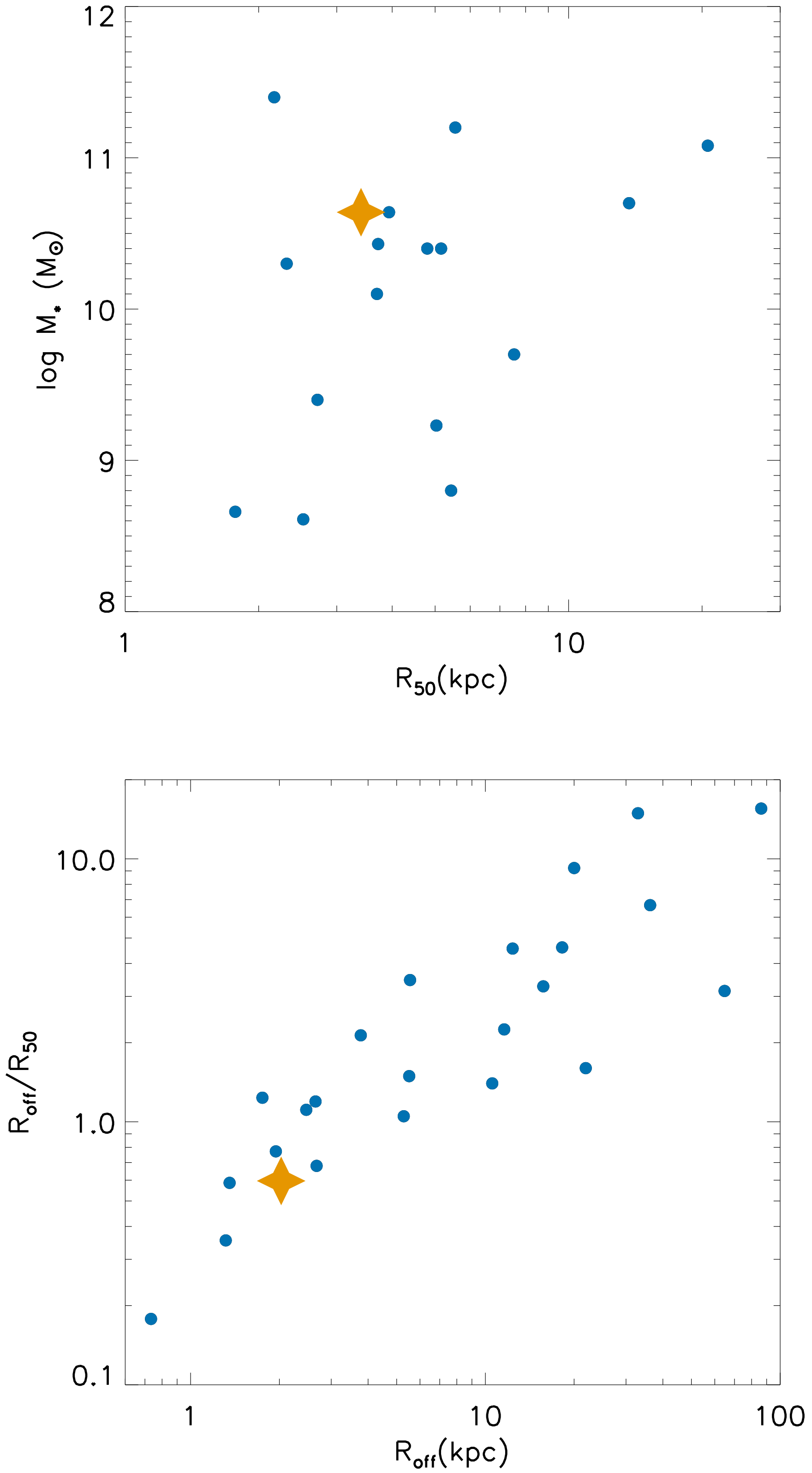

Supplementary Figure 7: *Top*: A comparison of the half light radius R_{50} and stellar mass M_* [34] between GRB 170817A and other sGRBs. *Bottom*: A comparison of the physical offset $R_{\rm off}$ and normalized offset $R_{\rm off}/R_{50}$ between GRB 170817A and other sGRBs. The dots indicates sGRBs in [2]. The orange star presents NGC 4993, which falls well into the distributions of the sGRB host galaxy properties.

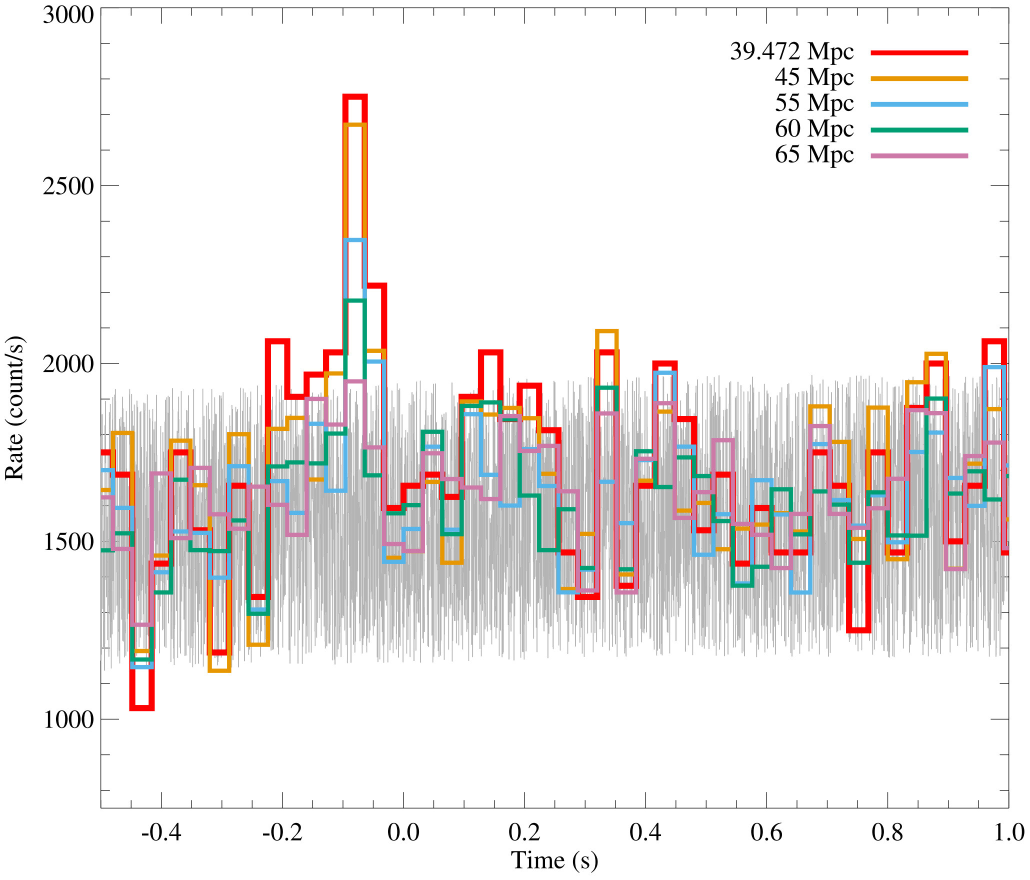

Supplementary Figure 8: GRB 170817A and simulated bursts by placing it at different distances. A 1-σ Poisson noise was added for each simulation. At 65 Mpc, the burst becomes not detectable.

Suppose that the GRB emission occurs at radius $R_{\rm GRB}$ with Lorentz factor Γ, the delay time from the launch of the jet to GRB emission is $t_{\rm prop} \sim R/2\Gamma^2 c$ [17]. The total delay time of the onset of the GRB with respect to the GW signal merger time would be

$$\Delta t \sim (t_{\rm prop} + \tau_{\rm acc} + \Delta t_{\rm jet})(1+z) \simeq (\tau_{\rm prop} + \Delta t_{\rm jet})(1+z). \tag{5}$$

An intriguing fact is that the GRB duration $T_{90} \sim 2$ s, which is similar to the delay time scale $\Delta t \sim 1.7$ s. If one does not introduce an ad hoc $\Delta t_{\rm jet} \sim 1$ s by hand (e.g. as invoked in the cocoon breakout model or the photosphere model [21]), one natural interpretation is that $\Delta t \sim t_{\rm prop} \sim T_{90} \sim R_{\rm GRB}/\Gamma^2 c$. Adopting $\Delta t = 1.7$ s from the data, the emission radius may be estimated as

$$R_{\rm GRB} \sim \Gamma^2 ct = 5 \times 10^{14}{\rm cm} \left(\frac{\Gamma}{100}\right)^2 \left(\frac{\Delta t}{1.7{\rm s}}\right) = 5 \times 10^{12}{\rm cm} \left(\frac{\Gamma}{10}\right)^2 \left(\frac{\Delta t}{1.7{\rm s}}\right), \tag{6}$$

which is usually much greater than the photosphere radius R_{ph}. The photosphere emission may give such a delay if $\Gamma < 5$. However, the temperature of the photosphere emission would be too low to explain the high value of $E_p \sim 158$ keV. If the emission is not from the photosphere, then the photosphere emission has to be suppressed via magnetization (e.g. [19]). One needs a Poynting flux dominated flow, advected to a large radius before magnetic dissipation happens (e.g. [20]). As a magnetic bubble penetrates throgh the surrounding cocoon, a jet structure naturally develops. An observer at a viewing angle $\theta_v \sim 28$ deg would observe a low-luminosity GRB with delay time scale comparable to the duration itself regardless of the unknown values of the Lorentz factor Γ and emission radius R.

Supplementary Note 9. Predicted afterglow properties

The interaction between the jet and its ambient medium could generate a strong external shock, where particles are believed to be accelerated, giving rise to broad-band afterglow emission [22]. According to standard afterglow models, the lightcurve for a given observed frequency (e.g. optical frequency $\nu_{\rm obs}$) could be calculated as

$$F_{t,\nu_{\rm obs}} = f(t, \nu_{\rm obs}; z, p, n, \epsilon_e, \epsilon_B, E_k, \Gamma_0), \tag{7}$$

where E_k is the isotropic kinetic energy of the jet, Γ_0 is the initial Lorentz factor of the jet and n is the interstellar medium (ISM) particle number density. ϵ_e and ϵ_B are the electron and magnetic energy fraction parameters, and p is the electron spectral index.

Based on the total emission energy of the prompt emission and assume a factor of 20% for the γ-ray emission efficiency, the kinetic energy of the jet E_k can be estimated as 1.83×10^{47} erg. For binary neutron star mergers, a low value for ambient medium density is usually expected, since they tend to have a large offset relative to the center of its host galaxy. Here we adopt the ambient medium

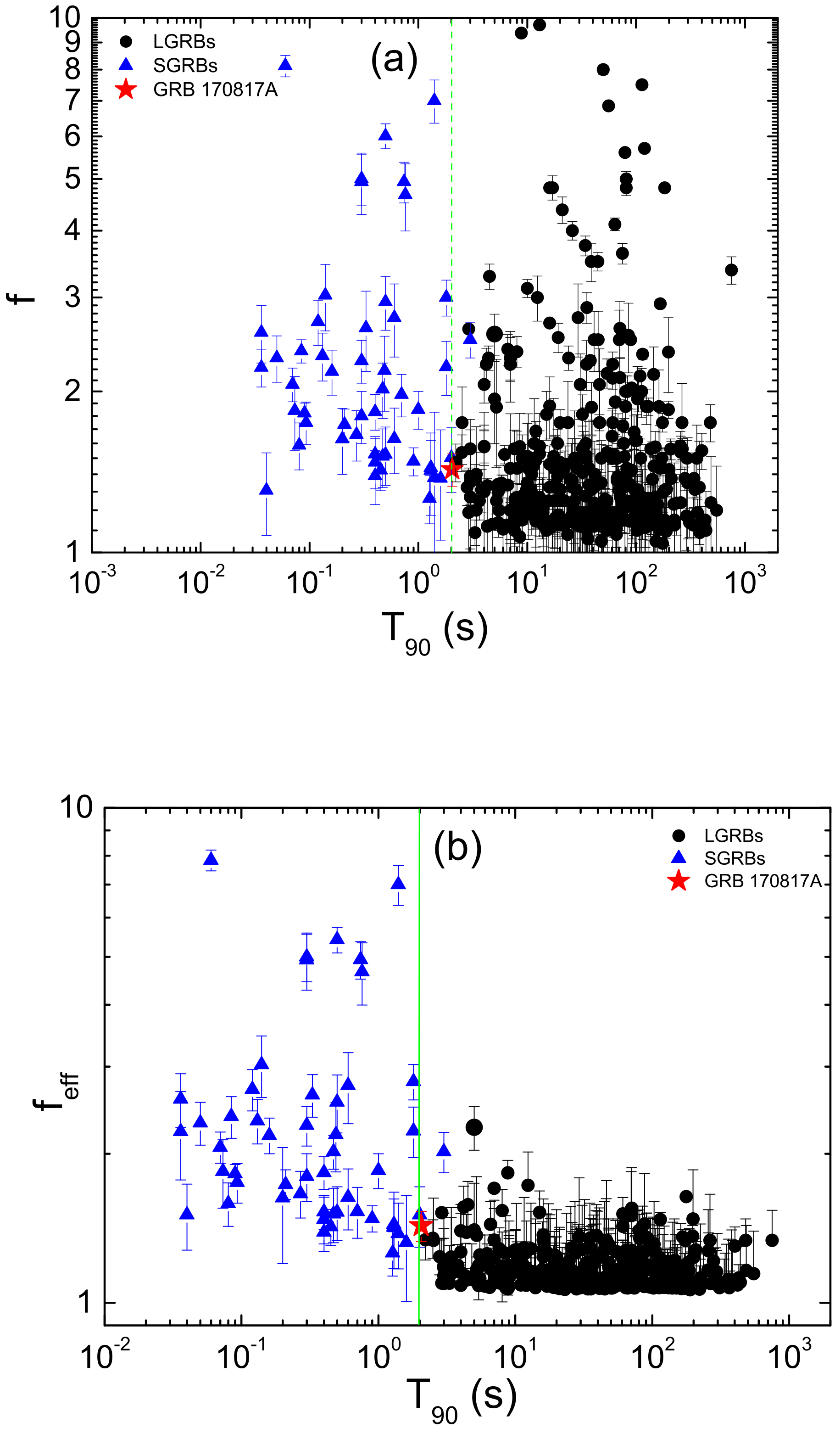

Supplementary Figure 9: T_{90} vs. f and f_{eff} diagrams of both long and short GRBs taken from [3]. The red solid star is GRB 170817, and the vertical line is $T_{90} = 2$ s. All error bars represent 1-σ uncertainties.

density n as 10^{-3} cm^{-3}. For a structured jet viewed from a large angle, the initial Lorentz factor may be low, so we adopt $\Gamma_0 = 20$. For other parameters, we adopt their commonly used values in GRB afterglow modeling, i.e., $\epsilon_e = 0.1$, and $p = 2.3$ [23, for a review]. The distribution of the ϵ_B value is wide, from $\epsilon_B = 0.01$ to $\epsilon_B < 10^{-5}$ [24–28].

We use $\epsilon_B = 0.01$ to calculate the most optimistic case of afterglow emission. The peak flux of the X-ray light curve emerges around 2000s, at the level of 10^{-14} erg/cm^2/s. Around 1 day, the X-ray flux will decay to the level of 10^{-15} erg/cm^2/s, under the detection limit of Swift/XRT. This is consistent with the non-detection result by the *Swift* team [29]. For optical band, the peak of the light curve appears around 2000 s, and the peak flux is 1 μJy (AB magnitude is 24). Around 1 day, the optical flux decays to the level of 0.01 μJy (AB magnitude is 28). This is much lower than the observed flux. This suggests that the optical transient detected by multiple groups originates from the emission of a kilonova [30–33]. The late emergence of the X-ray and radio emission [5, 6] are consistent with emission from the near-axis powerful jet being decelerated by the ambient medium [11].

Supplementary Note 10. Search for GRB 170817A-like events

Since it is possible that there are other GRB 170817A-like events in the GBM faint sGRB sample, we attempted to search for these events using the galaxy data. We choose all the faint bursts listed in Supplementary Figure 6, and look for NGC 4993-like galaxy (with luminosity 3.6×10^{43} erg s^{-1}) below 80 Mpc within the error boxes of the sGRBs. Supplementary Figure 10 shows the galaxies (red dots) with luminosity $> 10^{43}$ erg s^{-1} (left) and $> 3 \times 10^{43}$ erg s^{-1} (right) compared with sGRB error circles. It is clearly seen that the error circles are too large and typically enclose many galaxies. So identifying GRB 170817A-like events is difficult without gravitational wave detections.

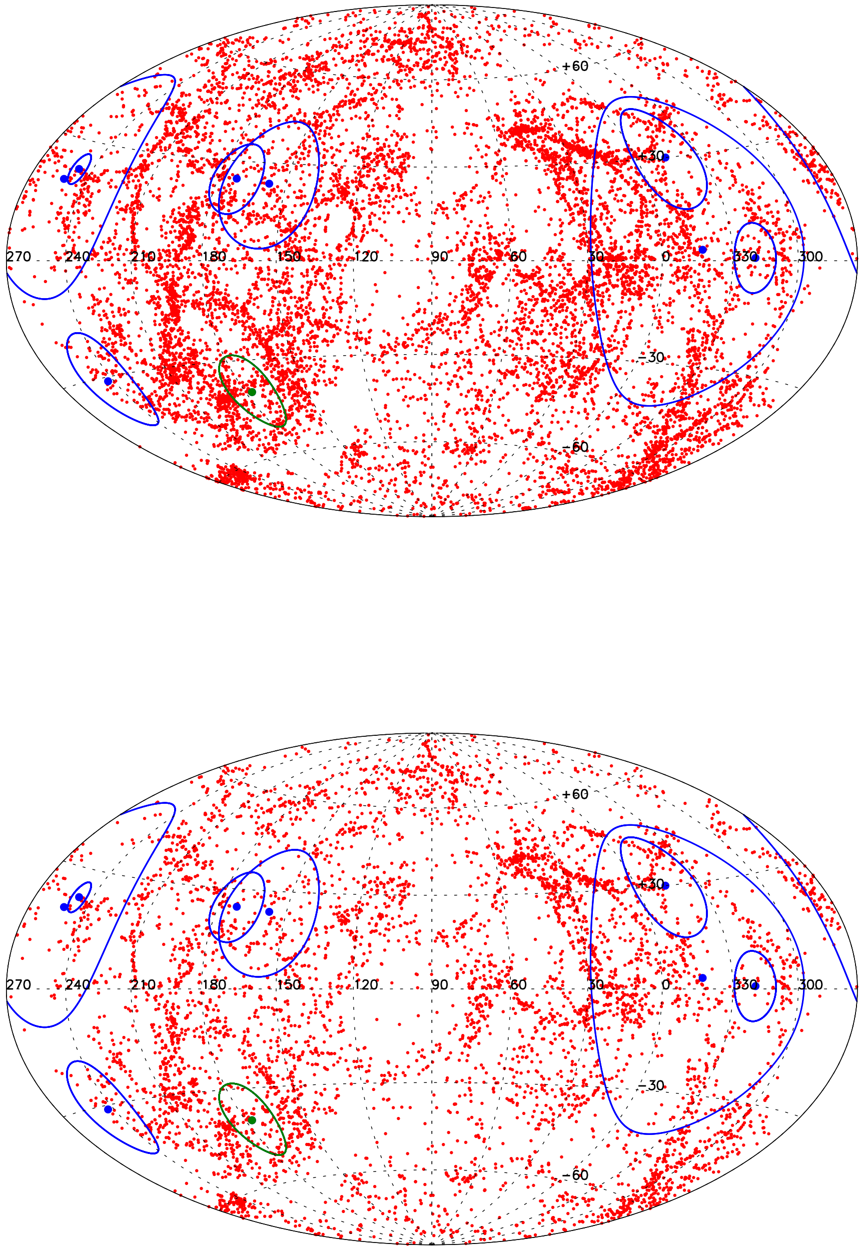

Supplementary Figure 10: The position error circles of GRB 170817A-like sGRBs in Figure 6 compared with the sky map of galaxies below 80 Mpc with two different luminosity threshold: $> 10^{43}$ erg s^{-1} (top) and $> 3 \times 10^{43}$ erg s^{-1} (bottom). The green color indicates GRB 170817A.

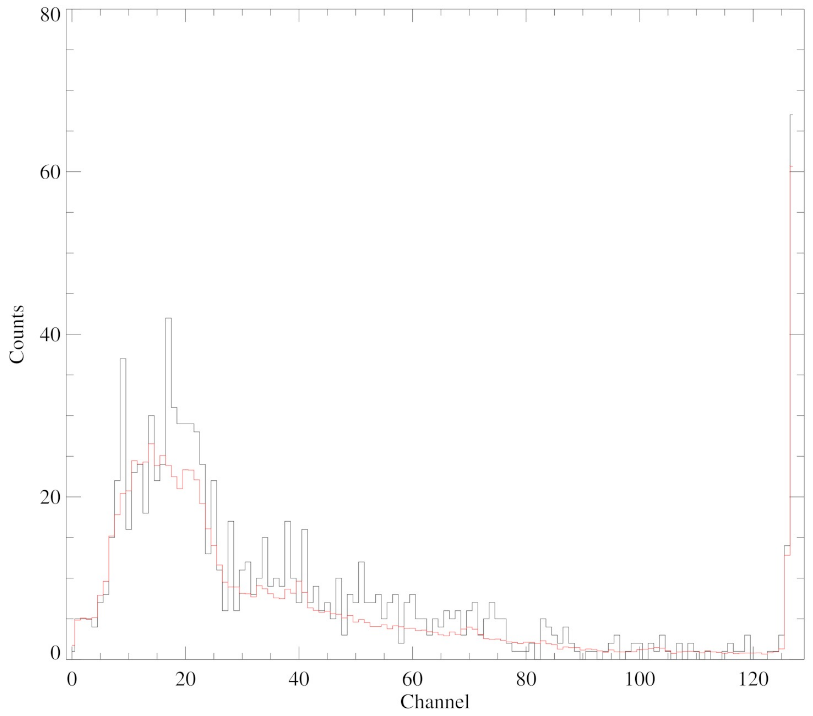

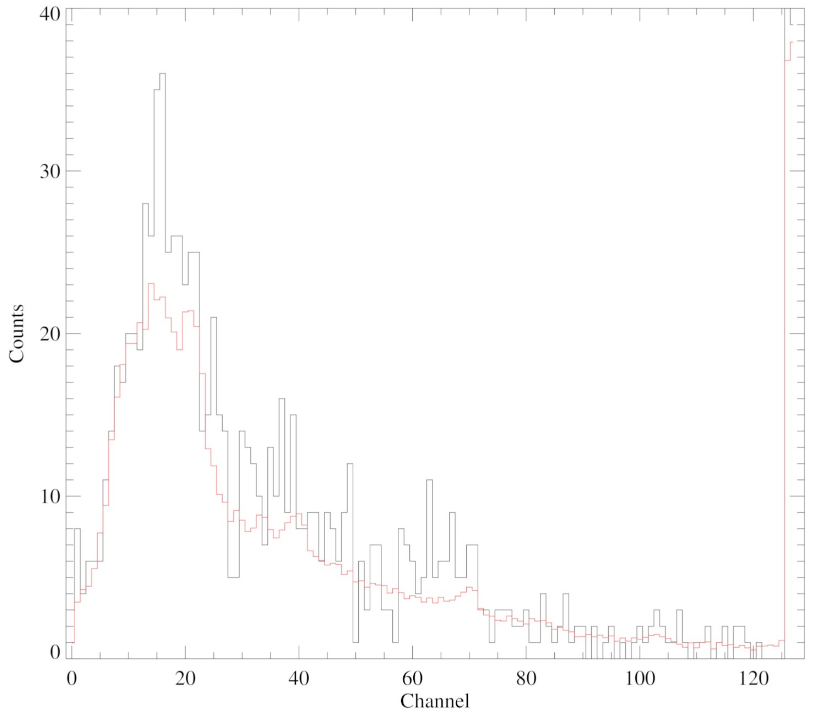

Supplementary Figure 11: Total counts (black) vs background counts (red) in each channel of the GBB NaI detectors in interval -0.26 to 0.57 s. Top: detector n1. The total counts over all the channels is 1048 , the total background counts over all the channels is 862. bottom: detector n2. The total counts over all the channels is 999 , the total background counts over all the channels is 822.

Supplementary References

1. Kass, R. E. & Wasserman, L., A reference Bayesian test for nested hypotheses and its relationship to the Schwarz criterion., *J. Am. Stat. Assoc.* **90**, 928-934 (1995)
2. Li, Y., Zhang, B., and Lü, H.-J., A Comparative Study of Long and Short GRBs. I. Overlapping Properties, *Astrophys. J. Suppl. S.* **227**, 7 (2016).
3. Lü, H.-J., Zhang, B., Liang, E.-W., Zhang, B.-B., & Sakamoto, T., The 'amplitude' parameter of gamma-ray bursts and its implications for GRB classification, *Mon. Not. R. Astron. Soc.*. **442**, 1922-1929 (2014)
4. Zhang, B., et al., Discerning the Physical Origins of Cosmological Gamma-ray Bursts Based on Multiple Observational Criteria: The Cases of z = 6.7 GRB 080913, z = 8.2 GRB 090423, and Some Short/Hard GRBs, *Astrophys. J.* **703**, 1696-1724, (2009)
5. Troja, E., Piro, L, van Eerten, H. et al., The X-ray counterpart to the gravitational-wave event GW170817, *Nature*, **551**, 71 (2017)
6. Hallinan, G., Corsi, A, Mooley, K. P. et al., A radio counterpart to a neutron star merger, *Science*, in press, Preprint at http://arxiv.org/abs/1710.05435 (2017)
7. Zhang, B. and Mészáros, P., Gamma-Ray Burst Beaming: A Universal Configuration with a Standard Energy Reservoir? *Astrophys. J.* **571**, 876-879 (2002).
8. Rossi, E., Lazzati, D., and Rees, M.J., Afterglow light curves, viewing angle and the jet structure of γ-ray bursts, *Mon. Not. R. Astron. Soc.* **332**, 945-950 (2002).
9. Lamb, G.P and Kobayashi, S., Electromagnetic Counterparts to Structured Jets from Gravitational Wave Detected Mergers, Preprint at http://arxiv.org/abs/1706.03000 (2017)
10. Lazzati, D., et al., Off-axis emission of short γ-ray bursts and the detectability of electromagnetic counterparts of gravitational-wave-detected binary merger, *Mon. Not. R. Astron. Soc.* **471**, 1652 (2017).
11. Xiao, D., et al., Afterglows and Kilonovae Associated with Nearby Low-Luminosity Short-Duration Gamma-Ray Bursts: Application to GW170817/GRB170817A, *Astrophys. J*, **850**, L41 (2017)
12. Abbott, B. P. et al. (LIGO Scientific Collaboration and Virgo Collaboration) Search for post-merger gravitational waves from the remnant of the binary neutron star merger GW170817, Preprint at http://arxiv.org/abs/1710.09320 (2017)
13. Zhang, B. and Mészáros, P., Gamma-Ray Burst Afterglow with Continuous Energy Injection: Signature of a Highly Magnetized Millisecond Pulsar, *Astrophys. J.* **552**, L35-L38 (2001).
14. Gao, H., Zhang, B., & Lü, H.-J., Constraints on binary neutron star merger product from short GRB observations, *Phys. Rev. D* **93**, 044065 (2016)
15. Piro, A.L., Giacomazzo, B., and Perna, R., The Fate of Neutron Star Binary Mergers, *Astrophys. J.* **844**, L19 (2017).
16. Yu, Y.-W. and Dai, Z.-G., A long-lived remnant neutron star after GW 170817 inferred from its associated kilonova, Preprint at http://arxiv.org/abs/1711.01898 (2017)
17. Zhang, B.: 2016, Mergers of Charged Black Holes: Gravitational-wave Events, Short Gamma-Ray Bursts, and Fast Radio Bursts, *Astrophys. J.* **827**, L31 (2016)
18. Mészáros, P., & Rees, M. J., Steep Slopes and Preferred Breaks in Gamma-Ray Burst Spectra: The Role of Photospheres and Comptonization, *Astrophys. J.* **530**,292-298 (2000)
19. Zhang, B., & Pe'er, A., Evidence of an Initially Magnetically Dominated Outflow in GRB 080916C, *Astrophys. J.* **700**, L65-L68 (2009)
20. Zhang, B., & Yan, H., The Internal-collision-induced Magnetic Reconnection and Turbulence (ICMART) Model of Gamma-ray Bursts, *Astrophys. J.* **726**,90 (2011)
21. Gottlieb, O., Nakar, E., & Piran, T. The cocoon emission - an electromagnetic counterpart to gravitational waves from neutron star mergers, *Mon. Not. R. Astron. Soc.* **473**, 576-584 (2017).
22. Gao, H., Lei, W.-H., Zou, Y.-C., Wu, X.-F., & Zhang, B., A complete reference of the analytical synchrotron external shock models of gamma-ray bursts, *New Astro. Rev.* **57**, 141-190 (2013)
23. Kumar, P., & Zhang, B., The physics of gamma-ray bursts & relativistic jets, *Phys. Rep.*. **561**, 1-109 (2015)
24. Santana, R., Barniol Duran, R., & Kumar, P., Magnetic Fields in Relativistic Collisionless Shocks, *Astrophys. J.* **785**, 29 (2014)
25. Wang, X.-G., et al., How Bad or Good Are the External Forward Shock Afterglow Models of Gamma-Ray Bursts?, *Astrophys. J. Suppl. S.*. **219**, 9 (2015)
26. Beniamini, P., Nava, L., Duran, R. B., & Piran, T., Energies of GRB blast waves and prompt efficiencies as implied by modelling of X-ray and GeV afterglows, *Mon. Not. R. Astron. Soc.* **454**, 1073-1085 (2015)
27. Zhang, B.-B., van Eerten, H., Burrows, D. N., Ryan, G. S., Evans, P. A., Racusin, J. L., Troja, E., & MacFadyen, A., An Analysis of Chandra Deep Follow-up Gamma-Ray Bursts: Implications for Off-axis Jets, *Astrophys. J.* **806**,15 (2015)
28. Ryan, G., van Eerten, H., MacFadyen, A., & Zhang, B.-B., Gamma-Ray Bursts are Observed Off-axis, *Astrophys. J.* **799**,3 (2015)
29. Evans, P. A. et al., *Swift* and *NuSTAR* observations of GW170817: detection of a blue kilonova, *Science*, doi:10.1126/science.aap9580 (2017)
30. Li, L.-X., & Paczyński, B., Transient Events from Neutron Star Mergers, *Astrophys. J.*. **507**, L59-L62 (1998)

31. Kulkarni, S. R., Modeling Supernova-like Explosions Associated with Gamma-ray Bursts with Short Durations, Preprint at http://arxiv.org/abs/astro-ph/0510256 (2017)
32. Metzger, B. D., et al., Electromagnetic counterparts of compact object mergers powered by the radioactive decay of r-process nuclei, *Mon. Not. R. Astron. Soc.* **406**, 2650 (2010)
33. Yu, Y.-W., Zhang, B., Gao, H., Bright "Merger-nova" from the Remnant of a Neutron Star Binary Merger: A Signature of a Newly Born, Massive, Millisecond Magnetar, *Astrophys. J.* **776**, L40 (2013).
34. Ogando, R.L.C., et al., Line Strengths of Early-Type Galaxies, *Astron. J.* **135**, 2424-2445 (2008).
35. Abbott, B. P. et al. (LIGO Scientific Collaboration and Virgo Collaboration) GW 170817: Observation of gravitational waves from a binary neutron star inspiral. *Phys. Rev. Lett.*, **119**, 161101 (2017).
