# Supplementary material for: A peculiar low-luminosity short gamma-ray burst from a double neutron star merger progenitor
Source: Nat Commun. 2018 Jan 31;9:447. doi: 10.1038/s41467-018-02847-3 (PMC5792494; doi:10.1038/s41467-018-02847-3)
Supplement: Supplementary file 2 — Peer Review File [file 41467_2018_2847_MOESM2_ESM.pdf]

Editorial Note: this manuscript has been previously reviewed at another journal that is not operating a transparent peer review scheme. This document only contains reviewer comments and rebuttal letters for versions considered at Nature Communications.
